# Characterizing axonal myelination within the healthy population: a tract-by-tract mapping of effects of age and gender on the fiber g-ratio

**DOI:** 10.1016/j.neurobiolaging.2016.09.016

**Published:** 2017-01

**Authors:** Mara Cercignani, Giovanni Giulietti, Nick G. Dowell, Matt Gabel, Rebecca Broad, P. Nigel Leigh, Neil A. Harrison, Marco Bozzali

**Affiliations:** a Department of Neuroscience, Clinical Imaging Sciences Centre, Brighton and Sussex Medical School, University of Sussex, Brighton, East Sussex, UK; b Neuroimaging Laboratory, Santa Lucia Foundation, Rome, Italy

**Keywords:** Myelination, White matter, Axonal conduction, Lateralization

## Abstract

The g-ratio, equal to the ratio of the inner-to-outer diameter of a myelinated axon, is associated with the speed of conduction, and thus reflects axonal function and integrity. It is now possible to estimate an “aggregate” g-ratio in vivo using MRI. The aim of this study was to assess the variation of the MRI-derived fiber g-ratio in the brain of healthy individuals, and to characterize its variation across the lifespan. Thirty-eight healthy participants, aged between 20 and 76, were recruited. Whole-brain g-ratio maps were computed and analyzed voxel-wise. Median tract g-ratio values were also extracted. No significant effect of gender was found, whereas age was found to be significantly associated with the g-ratio within the white matter. The tract-specific analysis showed this relationship to follow a nearly-linear increase, although the slope appears to slow down slightly after the 6th decade of life. The most likely interpretation is a subtle but consistent reduction in myelin throughout adulthood, with the density of axons beginning to decrease between the 4th and 5th decade.

## 1 Introduction

Myelin is a lipid-protein structure that wraps around axons, acting as an electrical insulator to increase the speed of action potential transmission. The amount of myelin wrapped around a nerve fiber determines its maximum conduction velocity. Initial observations in the peripheral nervous system (PNS), indicated that the myelin sheath thickness scales linearly with axon diameter (Berthold et al., 1983), prompting the concept of the “g-ratio”, equal to the ratio of the inner (axon only) to outer diameter (axon + myelin) of a nerve fiber. Models of axonal conductivity propose (Rushton, 1934) that the optimal g-ratio (maximizing fiber conduction speed) in the PNS is around 0.6. However, values measured in the central nervous system (CNS) have been consistently higher (Waxman and Bennett, 1972), necessitating a revision of Rushton's model of the optimal g-ratio to include additional factors that might influence its value in the CNS. When the simultaneous aims of reducing conduction delays, promoting conduction fidelity, lowering energy costs, and space saving are factored into this revised model, the theoretically optimal CNS g-ratio is calculated to be around 0.7 (Chomiak and Hu, 2009).

The tight empirical relationship between g-ratio and conduction speed suggests that g-ratio may represent a fundamental relationship between axonal anatomy and function. Region-specific changes in g-ratio have been described during neurodevelopment (Berthold et al., 1983) and may also provide a sensitive marker of pathology in a number of neurological conditions (Albert et al., 2007). The g-ratio is expected to relate the complex interaction of demyelination, remyelination, and axonal degeneration with neurophysiological abnormalities and thus with clinical symptoms. In particular, as the g-ratio can modify neuronal conduction velocity, it is conceivable that even subtle changes in g-ratio, likely to occur with aging, may mediate age-related cognitive decline.

Imaging (Bartzokis et al., 2012, Sullivan and Pfefferbaum, 2006, Yeatman et al., 2014) and histological (Almeida and Lyons, 2014, Nave and Werner, 2014) studies have confirmed that myelination changes throughout the lifespan. Myelin content increases in the first 2 or 3 decades of life, before reaching a plateau, then gradually reducing in the 5th to 8th decades (Yeatman et al., 2014). Interestingly, this degeneration does not occur uniformly across the brain but is typically faster for association tracts and more anterior white matter structures (Bartzokis et al., 2012, Pfefferbaum et al., 2000). Although an increase in the g-ratio is expected to parallel myelin reduction, the processes that regulate myelination in the adult brain are far from trivial (Nave and Werner, 2014), and direct investigations of g-ratio changes within the CNS have, to date, been difficult to implement.

Techniques to measure g-ratio in vivo have only recently become available. The first example was presented by Stikov et al. (2011), who combined fractional anisotropy (FA) from diffusion tensor imaging (DTI) and quantitative magnetization transfer (MT) to compute a mean voxel g-ratio in the corpus callosum. The same group later proposed a refined method (Stikov et al., 2015a), based on more sophisticated models of diffusion MRI (dMRI) than DTI, which can estimate a whole-brain “aggregate” g-ratio within a voxel. The term “aggregate” is used to indicate that the model assumes the g-ratio of all axons in an imaging voxel to be the same, and the resulting value is therefore a summary measure that does not provide any detail of the *distribution* of g-ratios within the voxel. The proposed model builds on simple geometric considerations and exploits the respective sensitivities of MT imaging to myelin, and of dMRI to intra-axonal water fraction. This method was validated against histology in the corpus callosum of the macaque brain (Stikov et al., 2015b), and further revised by West et al. (2016), who showed, using both theoretical considerations and histological validation, that the voxel-wise g-ratio estimated by MRI is equal to the root-mean-square of the g-ratios of individual axons weighted by their area. In other words, larger axons (which also tend to have higher g-ratios) will contribute more to the estimated mean g-ratio value than smaller axons.

This conceptual advance has opened the opportunity to use MRI to characterize whole-brain g-ratio in vivo. This is of particular importance since myelination varies considerably across tracts and varies markedly even within fibers belonging to the same tract (Tomassy et al., 2014) despite the optimal g-ratio expected in the CNS. This variability was confirmed by a recent paper (Mohammadi et al., 2015) providing the first g-ratio values estimated by MRI in 6 different white matter tracts from 37 young healthy participants. The results of this study, together with data from electron microscopy (Tomassy et al., 2014) suggest a poorly understood mechanism for the spatially-specific regulation of the myelination process. Furthermore, myelination of specific fibers has been shown to vary in a functionally-dependent manner (Scholz et al., 2009, Wang and Young, 2014).

The aim of this paper is to explore the anatomical distribution and the interhemispheric differences of the g-ratio in the healthy human brain and to investigate the dependence of the myelin volume fraction (MVF) and the axon volume fraction (AVF) on age and gender. In this way, we provide an interpretation of how myelin thickness and fiber density separately contribute to the observed variations in g-ratio mapping throughout the lifespan.

## 2 Methods

### 2.1 Participants

**Fig. 1:**
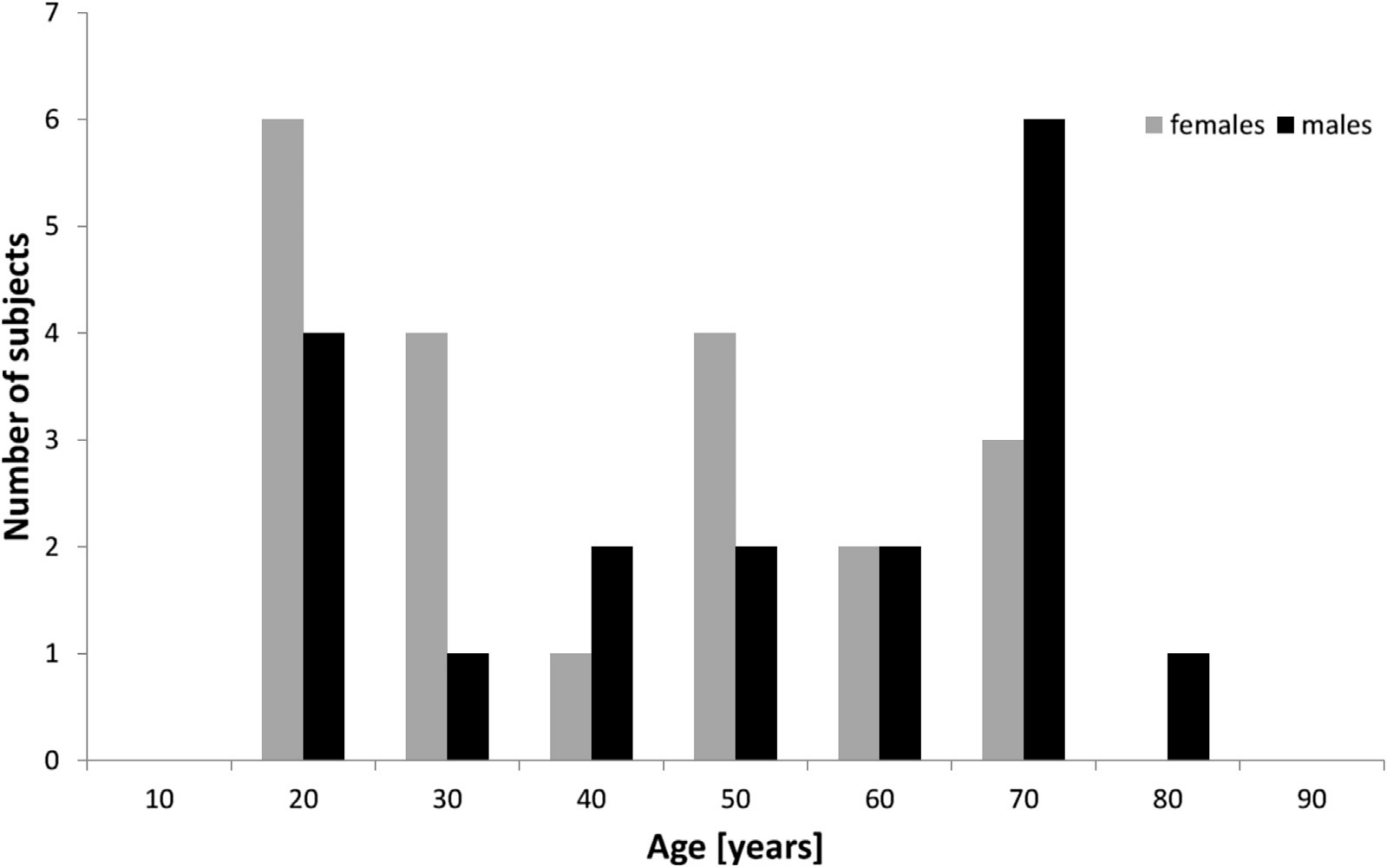
Age and gender distribution within the study cohort.

Thirty-eight right-handed healthy volunteers (18 males, median age: 45.5 years, range: 20–76 years) took part in the study after giving written informed consent. Participants were recruited through classified advertisements and university mailing lists. For a detailed distribution of age and gender, see Fig. 1. All participants were screened to exclude any neurological or psychiatric condition. The data were acquired as part of 2 studies approved, respectively, by the Herefordshire and by London and South East National Research Ethics Committees.

### 2.2 Magnetic resonance imaging

The 2 studies were run in parallel and MRI data were acquired on the same scanner, operating at 1.5 T (Siemens Magnetom Avanto, Erlangen, Germany), with a maximum gradient strength of 44mTm^−1^. The manufacture's 32-channel head coil was used for signal reception, whereas the body coil was used for transmission. A high-resolution T1-weighted volume (MPRAGE) was acquired for every participant.

Multi-shell diffusion-weighted (DW) MRI was acquired with single-shot, twice-refocused pulse-gradient spin-echo EPI (Reese et al., 2003), using 3 b values (9 directions with b = 300 smm^−2^, 30 directions with b = 800 smm^−2^, and 60 diffusion directions with b = 2400 smm^−2^), optimized for neurite orientation dispersion and density imaging (NODDI, [Zhang et al., 2012]). Ten nondiffusion–weighted (b = 0) volumes were also acquired. The remaining parameters were as follows: TE = 99 ms, TR = 8400 ms, matrix = 96 × 96, FoV = 240 × 240 mm^2^, slice thickness = 2.5 mm. The total scan time for the NODDI protocol was approximately 16 minutes.

Quantitative MT imaging was based on balanced steady-state free precession (bSSFP) model of MT, introduced by Gloor et al. (2008). The acquisition sequence consisted of a 3D True Fast Imaging with Steady-state Precession (TrueFisp) sequence (field of view = 240 mm × 180 mm, matrix = 256 × 96, slices = 32, slice thickness = 5 mm), modified to allow the duration of the radiofrequency pulse to be varied. Twenty-four volumes were acquired varying either the flip angle (between 5° and 40°) or the repetition time (between 3.66 ms and 5.96 ms) and the pulse duration. In addition, 3 three-dimensional fast low-angle shot (FLASH) volumes were acquired for T1-mapping, with repetition time = 30 ms and echo time = 5 ms. The excitation flip angles were varied between volumes (5°, 15°, 25°). The same field of view, matrix, and number of slices as the TrueFISP were used. The total scan time for the MT and T1 mapping data was ∼8 minutes.

### 2.3 Computation of parametric maps

DW MRI data were corrected for eddy current effects and involuntary movement by affine coregistration using the FLIRT tool (part of the FMRIB Software Library; FSL [Jenkinson et al., 2002]). Briefly, a within-b-value coregistration was performed, and average b = 0, b = 300, b = 800, and b = 2400 images were created. Each of these averages was coregistered to the mean b = 0 image to obtain the transformation matching them. Each DW volume was realigned to the mean DW image with the same b-value and the transformation matching each DW volume with the b = 0 image was obtained by combining the partial transformations from either previous steps. The b matrices were rotated accordingly (Leemans and Jones, 2009).

NODDI is an advanced model of diffusion which enables the estimation of 3 separate water compartments: intracellular, extracellular, and isotropic (i.e., CSF). The NODDI toolbox (http://www.nitrc.org/projects/noddi_toolbox) was used to fit the NODDI model to the movement-corrected data and to estimate the following metrics: neurite density index, corresponding to the intracellular volume fraction (v_ic_), orientation dispersion index, and isotropic volume fraction (v_iso_). The diffusion tensor and FA map were also estimated using the ordinary least-squares fitting in Camino (http://camino.cs.ucl.ac.uk/; Cook et al., 2006).

MT-bSSFP and T1 mapping volumes were spatially realigned to the 25° flip angle FLASH volume using rigid-body registration (FSL *flirt*, [Jenkinson et al., 2002]). T1 maps were obtained by fitting the 3 FLASH volumes to theoretical voxel values for the spoiled gradient echo for the 3 flip angles (Venkatesan et al., 1998).

The MT parameters forward exchange rate constant (k_f_), T_2_ of free water component (T_2f_), and bound proton fraction (*F*) were then obtained by performing a voxel-wise nonlinear least-squares fitting (Levenberg-Marquardt method) to a binary spin bath model (Gloor et al., 2008), using in-house software written in C.

To compensate for the spatial distortions in the DW EPI images, nonlinear warping implemented in Advanced Normalization Tools (ANTs; http://stnava.github.io/ANTs) was used to match the FA map to the 25° flip angle FLASH volume, to compute the transformation from DW-MRI to MT space. The same transformation was applied to the v_ic_ and v_iso_ map.

### 2.4 Computation of g-ratio

G-ratio maps were obtained as described by Stikov et al. (2015b). This model assumes the g-ratio to be constant within a voxel, which leads to the equivalence:

[1] $$g=\sqrt{1-\frac{MVF}{FVF}}=\sqrt{{\left(1+\frac{MVF}{AVF}\right)}^{-1}}\text{,}$$

where g is the g-ratio, MVF is the myelin volume fraction, FVF is the fiber volume fraction, and AVF is the axon volume fraction.

Following Stikov et al. (Stikov et al., 2015b), we set

[2] MVF=kF

and

[3] $$AVF=\left(1-MVF\right)\left(1-v_{\text{iso}}\right)v_{\text{ic}}\text{,}$$

where *k* is proportionality constant.

### 2.5 Estimation of proportionality constant *k*

The proportionality constant *k* in Eq [2] is not known a priori. It can vary depending on the specific MT method or model used (Stikov et al., 2015b) and requires histological validation. Previous attempts to estimate its value have led to inconsistent results (Dula et al., 2010, Thiessen et al., 2013).

Here we used a simple approach to derive *k* from a subsample of our data (Cercignani et al., 2016), similar to that used by Mohammadi et al. (2015). As the variation of g with age is not known a priori, we used only data from participants younger than 30 years of age (N = 17; M/F = 7/10; mean age = 25.7, standard deviation [SD] = 6.7 years).

Each participant's FA map (already coregistered with MT data) was nonlinearly coregistered (ANTs 1.9x) to the 2-mm^3^ isotropic resolution JHU FA template (Hua et al., 2008), available with FSL (http://fsl.fmrib.ox.ac.uk/fsl/fslwiki/Atlases). The same warps were applied to *F*, v_ic_ and v_iso_ maps. A figure demonstrating the goodness of registration achieved following this pipeline can be found as Supplementary Fig. 1. The JHU white matter tractography atlas was used to extract unbiased masks of the forceps major (thresholded at 20%). The mean *F*, v_ic_ and v_iso_ of this tract were estimated for every participant and then used to estimate the g-ratio for scaling factors *k* ranging from 1 to 5 and averaged across participant. The resulting g-ratio values were plotted against *k* to identify the study-specific value corresponding to g-ratio ≈ 0.7.

### 2.6 Standard space and tract-specific g-ratio

Once *k* has been estimated, we can compute Eqs [2], [3] voxel-wise, for each subject, using the coregistered v_ic_, v_iso_, and *F* maps.

To warp the g-ratio maps into standard Montreal Neurological Institute space for voxel-wise analysis, the same procedure described in the section “estimation of proportionality constant *k*” was followed for all participants.

The MPRAGE was segmented into white matter, gray matter, and CSF using the VBM8 pipeline (http://www.neuro.uni-jena.de/vbm/), and the white matter segment was thresholded at *p* > 0.5 and binarised. The resulting white matter masks were coregistered with the g-ratio maps in native space.

Finally, to obtain tract-specific median g-ratio values, 20 white matter tract masks were obtained from the JHU white matter tractography atlas (thresholded at 20% and binarized). The tracts contained in the atlas are: right and left anterior thalamic radiation, right and left cortico-spinal tract, forceps minor, forceps major, right and left cingulum bundles; right and left hippocampal portions of the cingulum; right and left inferior fronto-occipital fasciculi; right and left inferior longitudinal fasciculi; right and left superior longitudinal fasciculi; right and left temporal portion of the superior longitudinal fasciculi; left and right uncinate fasciculi. Using the inverse transformation obtained from the warping, the masks were transformed into each participant native space (MT space), combined with each participant's white matter mask (to remove any voxel falling outside of this tissue because of atrophy) and overlaid onto the coregistered g-ratio maps to extract the median values.

### 2.7 Statistical analysis

A voxel-wise analysis of g-ratio variation with age and gender was performed using a general linear model and estimating the *p*-values with permutation tests (with the FSL tool *randomize* [Winkler et al., 2014]). Two factors were modeled: gender and age. Initially, we also modeled the gender-by-age interaction. The analysis was restricted within the white matter, defined using the SPM8 white matter probability mask, thresholded at 0.6 and binarised. We used 2000 iterations, and the threshold-free-cluster-enhancement (TFCE [Smith and Nichols, 2009],) correction to account for multiple comparisons. Corrected *p*-values of less than 0.05 were accepted as statistically significant.

All the analyses of tract-specific g-ratio values were performed in SPSS (IBM SPSS Statistics for Windows, version 22.0. Armonk, NY, USA: IBM Corp). For each tract, an independent sample *t*-test was used to assess the effect of gender; the Pearson's correlation coefficient was used to assess the presence of associations between the g-ratio and age; whereas a paired-sample *t*-test was used to assess the interhemispheric difference. Bonferroni correction was used, and therefore we accepted as significant *p*-values <0.0025 for the independent sample *t*-tests and the correlations, and *p* < 0.005 for the paired-sample *t*-test.

To gain better insight into the variations occurring in g-ratio with age, we plotted the median values against age for each tract. To interpret what drives the variation in g-ratio throughout the lifespan, we also plotted the median tract values of both MVF and AVF against age. For each quantity, we compared 1st and 2nd order polynomial fits to assess which one better describes the data for each tract using the Bayesian Information Criterion (BIC, [Schwartz, 1978]), which chooses the best and most economical analytic model.

## 3 Results

All images were reviewed by an experienced observer to exclude the presence of any macroscopic abnormalities, and to qualitatively assess the raw data. In particular, MT-bSSFP images were inspected to evaluate the amount of banding artifacts; these were considered acceptable when restricted to the edges of the temporal lobe (which is the typical occurrence at 1.5 T). DW-MRI data were reviewed to exclude the presence of artifacts other than susceptibility-induced geometric distortions. None of the data sets were excluded based on these criteria. Among the older participants, 4 (2 males and 2 females, all older than 65 years) showed signs of minor small vessel disease, consistent with age.

### 3.1 Estimation of proportionality constant *k*

**Fig. 2:**
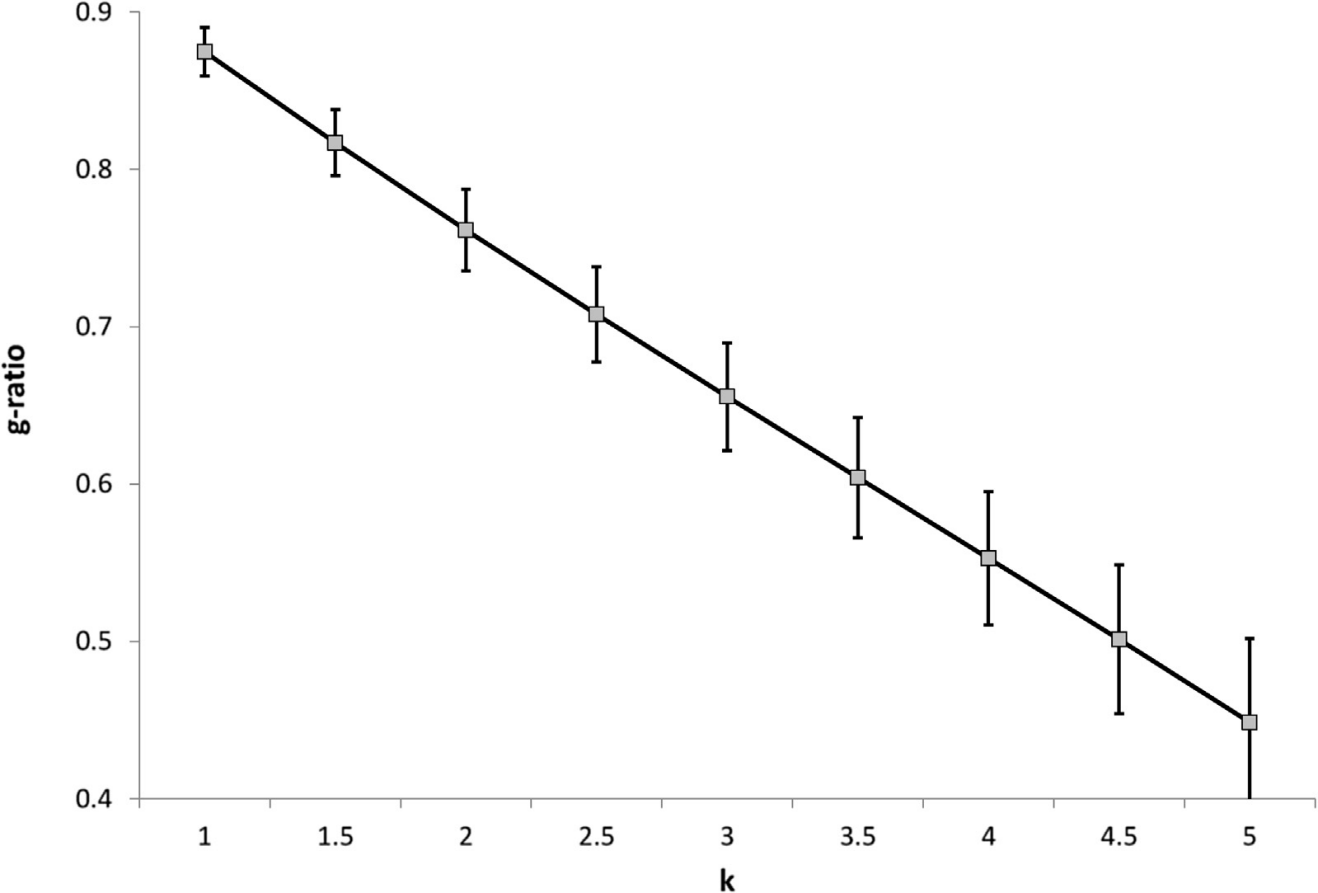
G-ratio estimated from the mean *F*, v_ic_, and v_iso_ of the forceps major. Values are averaged across subjects and plotted against *k*. The bars indicate the cross-subject standard deviation.

Fig. 2 shows the simulated g-ratio values for the forceps major. These data suggest that a value of *k* = 2.5 provides a g-ratio of ∼0.7 for our data. This value was used for all the subsequent calculations based on Eq [2].

### 3.2 Voxel-wise analysis

**Fig. 3:**
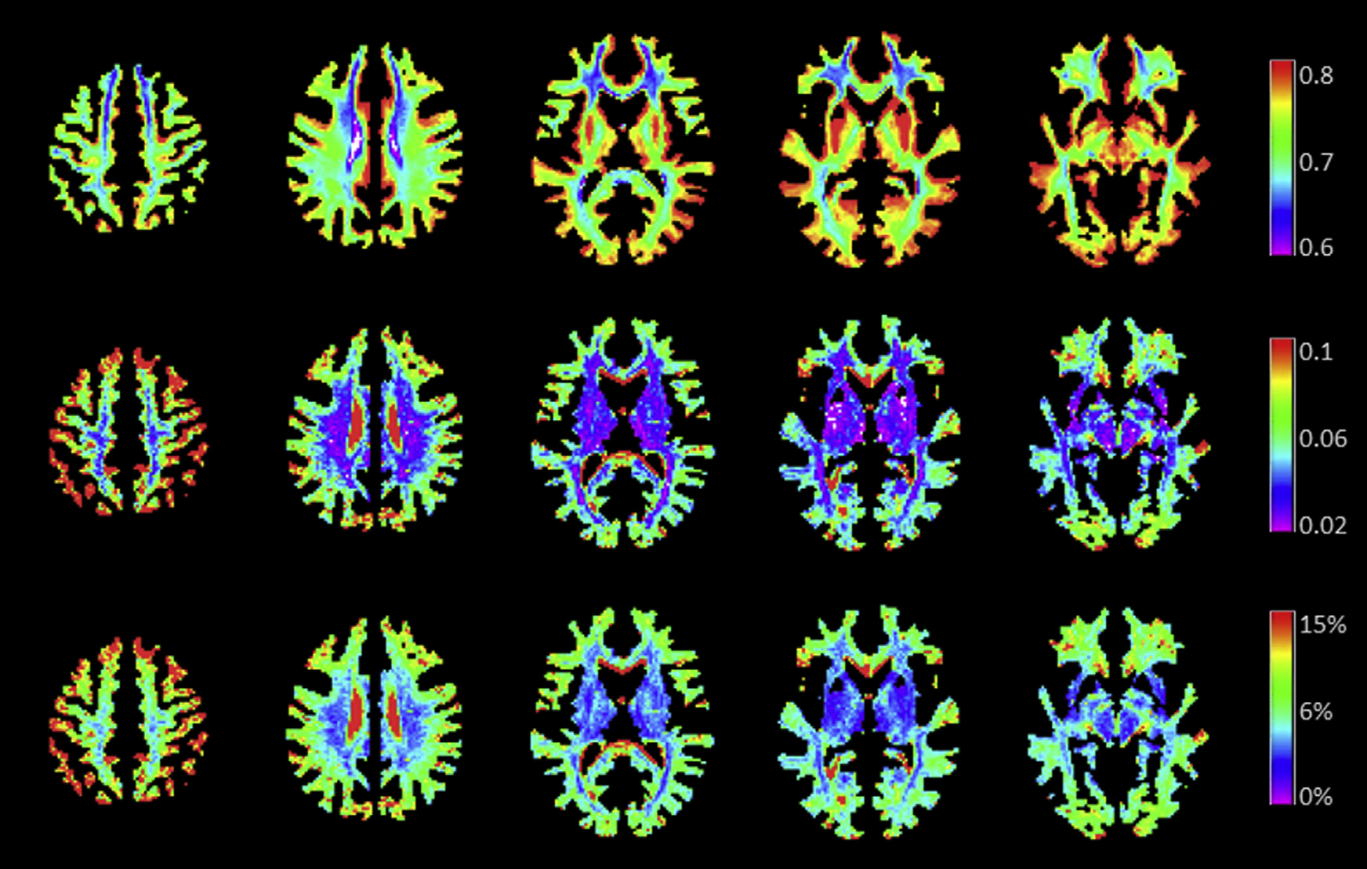
Voxel-wise maps of the mean (top), standard deviation (middle), and coefficient of variation (CoV, bottom) for the g-ratio computed across participants. The mean value in the white matter is in agreement with the expected value of 0.7. The inter-subject CoV is very low in the white matter (<8%).

Fig. 3 shows maps of the mean, SD, and coefficient of variation (SD/mean%) of the g-ratio across all 38 participants. These data confirm that the estimated g-ratio in white matter lies predominantly between 0.6 and 0.8, with 0.7 being the most represented value. The very low CoV (<10% in most of the white matter) also indicated good reproducibility and excellent between-subject alignment (within the white matter).

No significant age-by-gender interaction was found anywhere in the brain, therefore we removed the interaction term from the model to assess between-gender differences and the association with age.

**Fig. 4:**
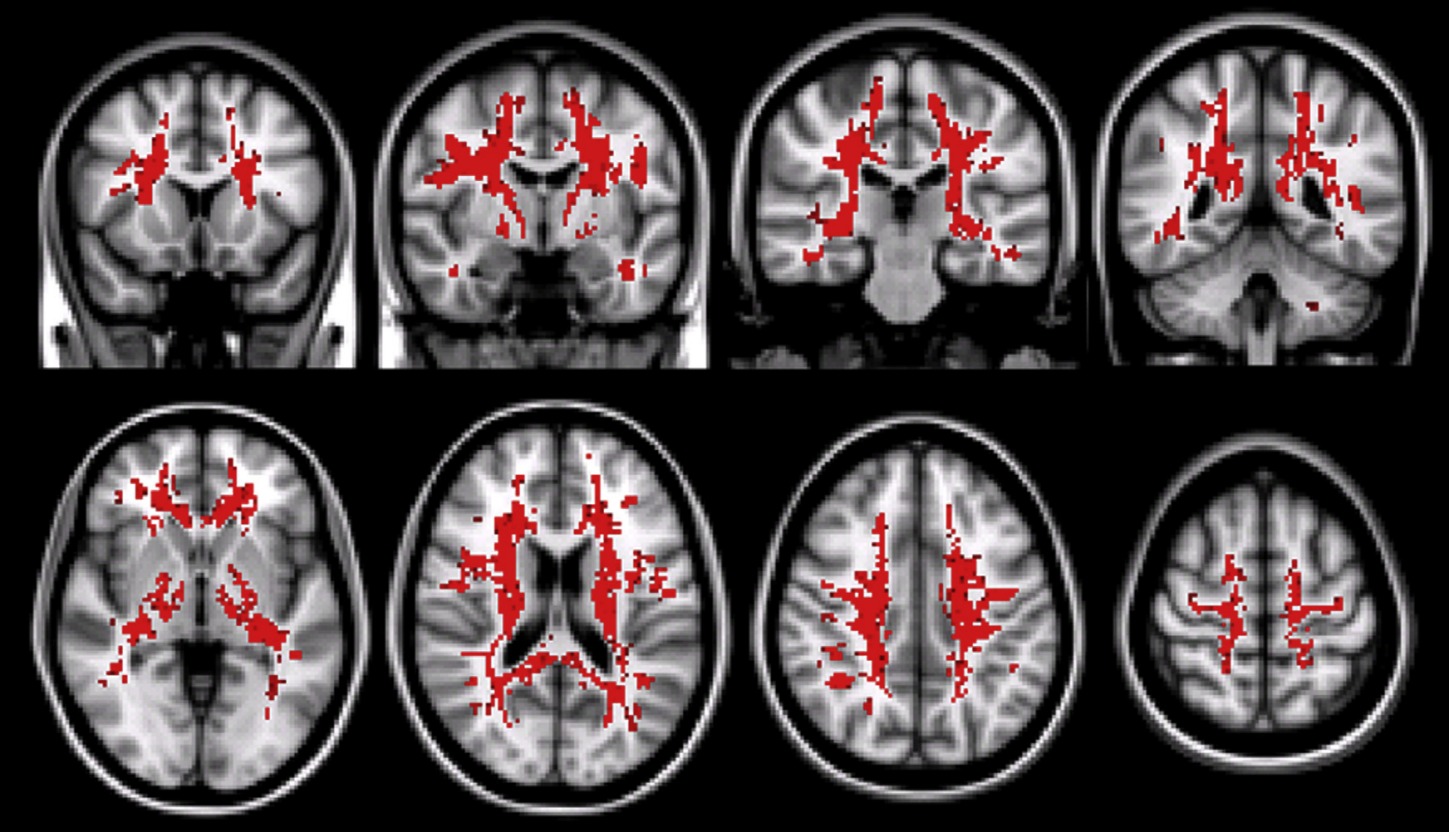
Results of the voxel-wise correlation between g-ratio and age. Voxels in red indicate positive association, with *p* < 0.05, corrected for multiple comparisons using the TFCE method. The analysis was restricted to the white matter. Abbreviation: TFCE, threshold free cluster enhancement. (For interpretation of the references to color in this figure legend, the reader is referred to the Web version of this article.)

No significant effect of gender was found. By contrast, age was found to be significantly associated with the g-ratio almost everywhere within the white matter (Fig. 4).

### 3.3 Tract-specific analysis

The mean, standard deviation, and range of tract volume after warping into native space and combination with white matter masks are reported in Table 1.

**Fig. 5:**
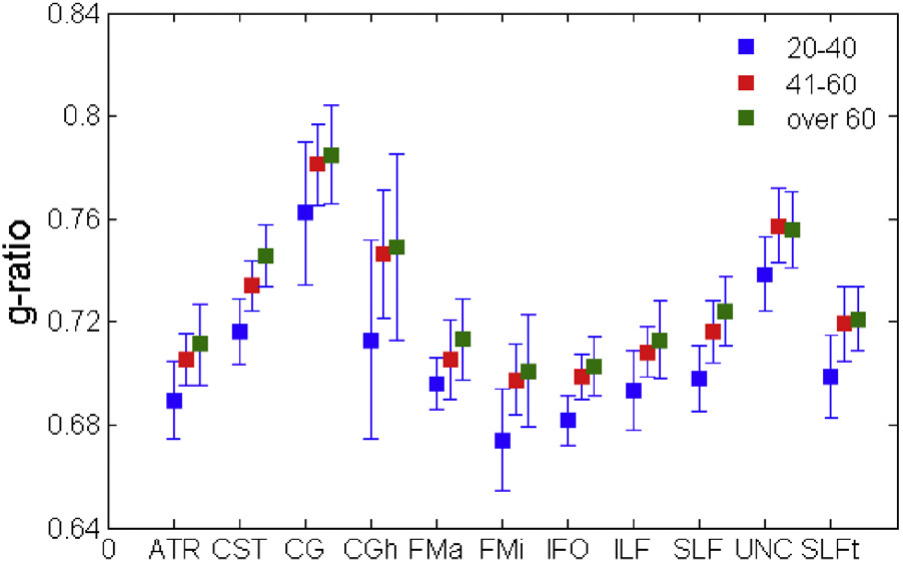
Comparison of mean g-ratio across all the white matter tracts studied, for participants grouped in 3 age groups. Values of left and right were pooled for bilateral tracts. Abbreviations: ATR, anterior, thalamic radiation; CST, cortico-spinal tract; ILF, inferior longitudinal fasciculus; SLF, superior longitudinal fasciculus.

Consistent with the voxel-wise analysis, no gender effect was observed for the g-ratio in any tract, with the exception of the left ILF, where females had higher mean g-ratio (0.711) than males (0.694), with *p* = 0.002. Fig. 5 shows the median g-ratio per tract, separately for 3 age groups: 20–40 (younger), 41–60 (middle-aged), and over 60 (older). For the purpose of this figure data from the left and right hemisphere were pooled. No tract showed a median g-ratio lower than 0.67. The younger group had a lower g-ratio than the other 2 groups in all tracts, with the lowest values found in the forceps minor (0.671), and the largest in the cingulum bundle (0.758). Differences between the middle-aged and the older groups were less pronounced and less systematic than those observed between the younger and the middle-aged groups.

G-ratio was significantly correlated with age for each of the tracts studied (*p* < 0.05), with the exception of the right cingulum (*p* = 0.1). These correlations remained significant after Bonferroni correction (*p* < 0.0025) with the exception of the right inferior longitudinal fasciculus, the uncinate fasciculus (bilaterally), and the hippocampal portion of the cingulum bundle (bilaterally).

**Fig. 6:**
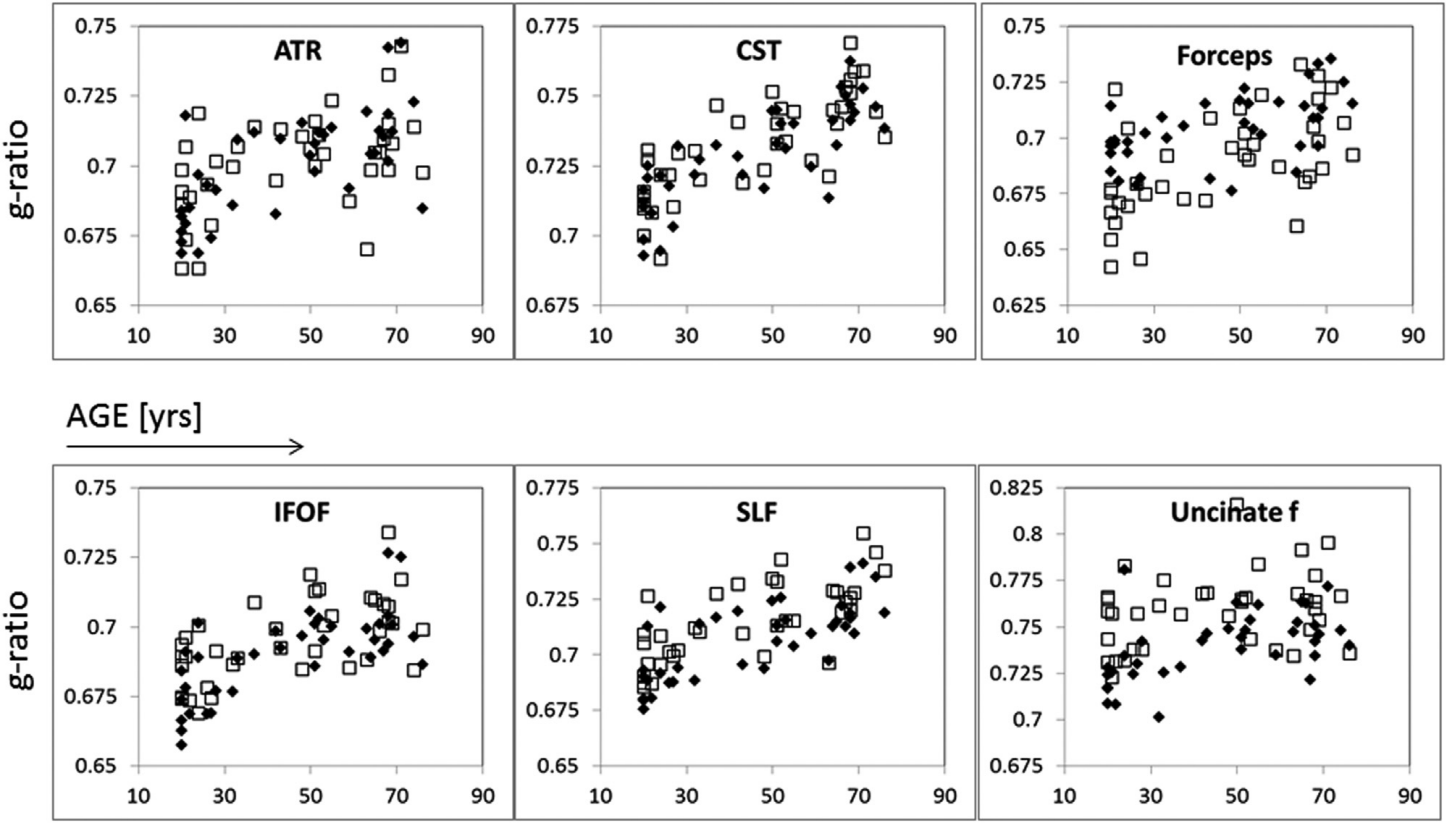
Scatterplots of median tract g-ratio versus age for 6 representative tracts out of the 10 reconstructed. Data for the right (gray squares) and left (black diamonds) hemisphere are shown, with the exception of the forceps, where gray square represent data from the forceps minor and black diamonds represent data from the forceps major. Abbreviations: ATR, anterior thalamic radiation; CST, cortico-spinal tract; IFOF, inferior fronto-occipital fasciculus; SLF, superior longitudinal fasciculus.

When plotting median g-ratio against age, some tracts showed a linear increase with age (Fig. 6, e.g., the cortico-spinal tract and the SLF). Others showed a more quadratic dependency with the aggregate g-ratio increasing between 20 and 60 years of age then reaching a plateau (Fig. 6, e.g., IFOF). Finally, for some tracts, such as the cingulum bundle, no obvious dependency could be observed. Nevertheless, the results of the BIC comparison indicate that the linear and quadratic models perform equally in most of the tracts.

Significant (*p* < 0.05) inter-hemisphere differences were observed for most tracts with the exception of the cingulum bundle, the hippocampal part of the cingulum bundle, the anterior thalamic radiation, and the inferior longitudinal fasciculus. The laterality effect remained significant (*p* < 0.005) after Bonferroni correction for the uncinate fasciculus, the SLF, the inferior fronto-occipital fasciculus, and the cortico-spinal tract, with higher values in the right compared to the left (dominant) hemisphere. Laterality seemed to be maintained throughout the life span (see ATR and uncinate fasciculus in Fig. 6).

**Fig. 7:**
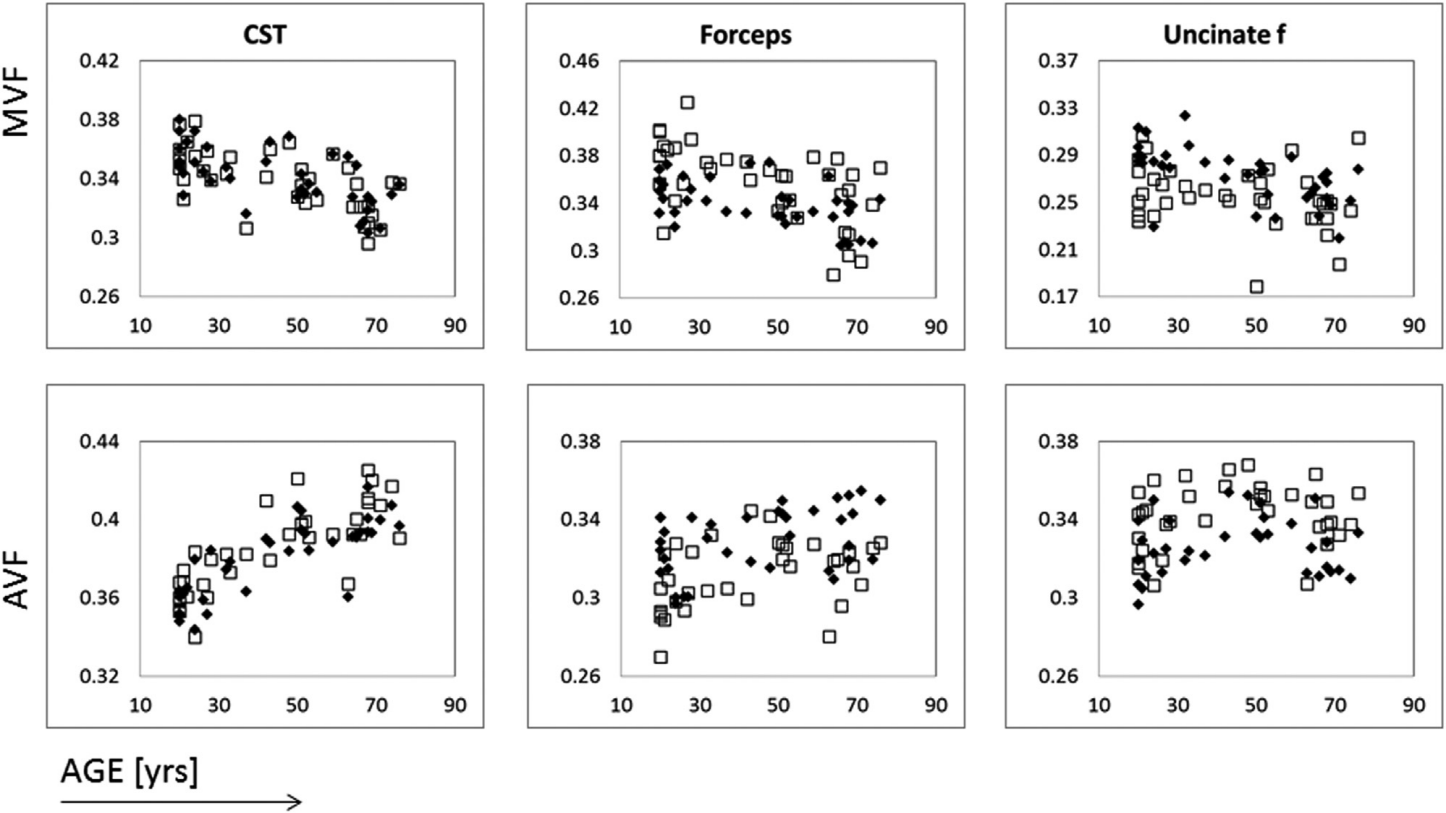
Scatterplots of median tract myelin volume fraction (MVF, top) and axon volume fraction (AVF, bottom) versus age for 3 representative tracts out of the 10 reconstructed. Data for the right (gray squares) and left (black diamonds) hemisphere are shown, with the exception of the forceps, where gray square represent data from the forceps minor and black diamonds represent data from the forceps major. Abbreviation: CST, cortico-spinal tract.

When we looked at the variability with age of MVF and AVF, we observed that MVF tends to decrease with age in most tracts (see Fig. 7, top panel). The variations in AVF were more tract-specific. In some tracts, AVF appeared relatively stable across the lifespan (e.g., Forceps major and forceps minor). However, in other tracts it appeared to increase to approximately 50 years of age then decrease (e.g., ATR, IFOF, ILF, and uncinate fasciculus). In a few other tracts, the increase in AVF appeared to remain constant until very late in life (e.g., CST, SLF, cingulate bundle). Fig. 7 shows 3 tracts as representative examples. Again, we compared linear and 2nd-order polynomial fits using the BIC, which indicates that the linear model is to be preferred for MVF in all tracts. Conversely, for AVF, the quadratic model performs better in the anterior thalamic radiations, the inferior fronto-occipital fasciculus, the inferior longitudinal fasciculus, the SLF, and the forceps minor.

## 4 Discussion

Here we present a detailed investigation of the factors affecting the variability of the g-ratio within the CNS. The main findings can be summarized as follows: gender has no influence on regional g-ratio; age significantly modulates the g-ratio; and there is evidence for g-ratio laterality in some association tracts. In addition, we showed that substantial g-ratio variability exists within the brain.

### 4.1 G-ratio dependency on gender

The lack of evidence for a gender-dependent g-ratio difference might be surprising, in light of the previously-reported sex differences in the volume and microstructure of the white matter (Good et al., 2001, Hsu et al., 2008). Gender differences within the CNS are known to occur during neurodevelopment (Perrin et al., 2009), and a between-gender shift in the g-ratio has previously been proposed as a possible explanation for the emergence of sex differences during adolescence (Paus and Toro, 2009). Our data, by contrast, indicate that g-ratio is not significantly influenced by sex, at least not in adults. This result should be interpreted with caution, as the sample size was relatively small, and, admittedly, male participants were on average slightly older than females in our sample, but this difference was not statistically significant (independent sample *t*-test, *p* = 0.13). Furthermore, when the gender-by-age interaction was incorporated into the model it did not change our findings. We speculate that the absence of a gender effect on g-ratio does not imply that such an effect does not exist for the absolute degree of myelination, but that the relationship between inner and outer axonal diameters is not gender dependent. This is conceivable in light of its functional interpretation, that is, being a determinant of speed of conduction.

### 4.2 G-ratio dependency on age

By contrast, age has a very significant effect on the g-ratio: this was evident from both voxel-wise and the tract-specific analysis. This result is in keeping with previous reports indicating changes in the white matter with aging (Sullivan and Pfefferbaum, 2006) and indicates that the g-ratio increases with age, consistent with the expected thinning of the myelin sheath (Peters, 2009). While specific changes in the g-ratio during the lifespan have never been investigated in the human brain, histological data from the sciatic nerve (Ugrenovic et al., 2016) indicate that in the PNS the percentage of myelinated nerve fibers with large diameter and optimal g-ratio declines significantly with age. As demonstrated by West et al. (2016), MRI provides a g-ratio estimate that is an average across the whole voxel, weighted by the axonal area. Consequently, changes to this quantity could reflect not only global changes in the inner-to-outer axonal diameter ratio but also a change in the distribution of g-ratios within the voxel, and the balance between small- and large-diameter axons. Fibers with smaller diameters tend to have slightly lower g-ratios (Berthold et al., 1983), and therefore a selective loss of large-diameter axons would lead to an overall reduced voxel-averaged g-ratio. Based on the data we have, however, we can only conclude that the aggregate g-ratio tends to increase with age throughout the brain, and histological validation would be required to provide a definite interpretation of these findings.

Nevertheless, further information can be gathered from the analysis of MVF and AVF, and of their variability with age. We observed almost monotonic decreases in MVF, although the trend was different for different tracts. The BIC comparison confirmed that a 1st-order model better describes MVF variability with age than a 2nd-order one. This finding is in contrast with the observations of Yeatman et al. (2014), who used R1 (= 1/T1) as a proxy for myelination in a group 102 participants, aged 7 to 85. They found an inverted U-shaped trend which was symmetric around the peak. It is unclear why we were not able to reproduce their findings; however, it is possible that, not having data for subjects younger than 20, given the variance around the mean, and the relatively small sample size, we are simply unable to capture the initial increase in MVF. Interestingly, AVF seems to vary with age in a nonlinear fashion (at least for some tracts). As expected, AVF tends to decrease after the age of 50 for most tracts, but before that age we observed an apparent increase. Previous studies based on imaging have shown nonlinear changes in diffusivities, fractional anisotropy, and white matter volume (Walhovd et al., 2014). While a direct comparison between those data and AVF is not possible, evidence from volumetric studies suggests that white matter volume as a whole peaks around 40 years of age (Westlye et al., 2010). We could therefore conclude that the observed variation in AVF results from the complex sequence of maturation and aging processes in the white matter.

White matter changes with aging have been amply demonstrated using a range of imaging techniques, including DTI and relaxometry (Billiet et al., 2015), all supporting the hypothesis that some white matter structures are more vulnerable than others to the aging process. One of the theories developed to explain this phenomenon is the so-called retrogenesis hypothesis (Brickman et al., 2012), which postulates that late-myelinated white matter fibers are most vulnerable to age- and disease-related degeneration. While our data suggest greater myelin density in the CST and corpus callosum as opposed to the UF and ILF, they do not support the hypothesis that the loss of myelin in the latter tracts is faster or onsets earlier than in the former ones. We are not the first to fail to confirm this theory, with some previous studies even suggesting an increase of myelination with age (Billiet et al., 2015).

While no previous study investigated g-ratio variations with age in adults, 2 previous reports looked, respectively, at changes in infants (aged 3 months to 7 years; Dean et al., 2016) and preterm babies (Melbourne et al., 2014). As expected, the g-ratio was found to be approximately 1 (no myelin) at very early ages, sharply decreasing during the first 2 years of life, to follow a slower decrease later on toward an asymptotic value of 0.7–0.8. Interestingly, Dean et al. (2016) found the asymptotic value to differ for distinct white matter regions, with the cingulum, the SLF, and the internal capsules reaching higher values than the corpus callosum. This is in line with our findings of higher g-ratio in young adults in the cingulum and in the SLF, compared to the forceps.

### 4.3 Anatomical distribution of g-ratio

Despite the relatively homogeneous rate-of-variation of the g-ratio along the lifespan, our data confirm a substantial anatomical variability of this parameter (see Fig. 5), even in young participants. This fits with the preliminary observations by Mohammadi et al. (2015). When comparing our tract-specific estimations of the g-ratio with theirs, however, some important differences appear. They found the cingulum bundle, the optic radiation, and the IFOF to have significantly lower g-ratios than other tracts such as the CST, the fornix and the optic radiation. While we looked at a slightly different sample of tracts, we found the cingulum to have the highest g-ratio, in clear contradiction with the previous report. We can only speculate of what might be the origin of this disparity, probably related to methodological considerations. First, we used a different white matter tract atlas, and different thresholds (we retained all voxels with a probability of greater than 20% of being part of the tract, while they only used those with probability higher than 50%). When computing the mean/median over the whole tract, it is possible that voxels in the “periphery” (which are likely to have been included in larger numbers here than in the previous study) contribute higher g-ratio values than the ones in the core. Another, perhaps more important, observation is that we used Stikov's original model of the g-ratio (Stikov et al., 2015a), while Mohammadi et al. introduced an alternative method, where the fiber volume density is estimated based on an index called the tensor fiber density (Reisert et al., 2013). While this latter method has the advantage that it can be derived from single shell dMRI data, it is also known to show very high-spatial variability (Reisert et al., 2013). In addition, because of the low b-value used for their acquisition (b = 1000 mm^2^s^−1^) the tensor fiber density can be affected by contributions from the extracellular water (Mohammadi et al., 2015). Furthermore, the myelin signal contributing to dMRI is negligible even in the presence of significant myelin volume due to the long echo times associated with dMRI and the comparatively-short T2 of myelin. Importantly, Stikov's model explicitly takes this into account and accordingly corrects the AVF, and thus the FVF, whereas the tensor density approach does not. All these factors are likely to affect the final g-ratio estimation, and only detailed validations in animal models will be able to conclusively determine the accuracy of either method.

### 4.4 Estimation of the proportionality constant

Another important limitation, which is common to both methods is the need for the estimation of an arbitrary proportionality constant (*k* in our case), which could potentially introduce some bias. We followed an approach similar to that of Mohammadi et al. (2015), and derived *k* for the fibers originating from the splenium of the corpus callosum (forceps major), where the g-ratio is expected to be approximately equal to 0.7 (Stikov et al., 2015b). Despite this procedure may be seen as arbitrary, the value we estimated for *k* of 2.5 is consistent with the value originally derived by Dula et al. (2010) from histological validation in the spinal cord white matter. It is however important to highlight that a wrong calibration could lead to the computed g-ratio having some dependency on FVF (Campbell et al., 2016). While the resulting quantity is still weighted by the aggregate g-ratio, caution must be used when interpreting changes to it.

### 4.5 Laterality of g-ratio

It is important to reiterate that, while the g-ratio is a fundamental quantity, the MRI-estimated g-ratio is a new concept and warrants further work toward its full validation. It is possible that problems and limitations of this technique will only become evident as we continue to develop its applications. Nevertheless, the noninvasive estimation of this parameter opens exciting new possibilities in establishing structure-function relationships within the CNS.

Indeed, we reported that while interhemispheric differences are negligible for most white matter tracts, some association tracts, namely the uncinate fasciculus, the SLF, the inferior fronto-occipital fasciculus, and the cortico-spinal tract, show a very significant laterality effect. Left-to-right asymmetries in the association bundles have been reported previously with other methodologies (e.g., [Takao et al., 2011]), and are believed to be related to specialized functions of the brain, such as language, which are lateralized in humans (Parker et al., 2005). Some authors have even suggested a potential link between the absence of asymmetry and psychosis (Park et al., 2004). Although it is unclear what determines this spatially-specific change in myelin thickness, a fascinating hypothesis linking it to function postulates that myelination is at least in part regulated by neuronal activity (Lundgaard et al., 2013, Scholz et al., 2009, Wang and Young, 2014). Interestingly, we observed lower g-ratio values in the left (dominant) hemisphere. It is unfortunately not possible to explore any relationship with handedness in this sample, as all participants were right handed. Assessing the relationship between the estimated g-ratio values and the performance at specific tasks testing lateralized functions could perhaps support this intriguing suggestion; however, we did not collect cognitive data in this sample.

### 4.6 White matter plasticity

As a final remark, we observe that if “myelin plasticity” exists (Wang and Young, 2014), it is very likely that each individual's g-ratio is not only affected by easily accountable factors such as age but also by a collection of personalized factors that depend on their education and lifestyle, cognitive stimulation, and physical experiences. Studies in rodents have indicated that physical exercise (Krityakiarana et al., 2010) and socialization (Liu et al., 2012) promote white matter plasticity. In this view, a limitation of our study is the lack of supporting data on an individual's lifestyle and could justify the large variance we observe around the average trend with age (Fig. 6). Future work should address this interesting topic and establish the relationship between the g-ratio and factors that can promote neural activity and therefore increase myelin production via modulation of oligodendrocyte progenitor cells proliferation (Wang and Young, 2014).

### 4.7 Methodological considerations

It is important to reiterate that the aggregate g-ratio estimated by MRI relies on a number of assumptions with respect to the 2 underlying techniques, MT and NODDI. Both methods suffer from some limitations and any error to the parametric maps derived from them will of course propagate into the g-ratio. While *F* has been shown to correlate with myelination (e.g., [Turati et al., 2015]), MT is also sensitive to other factors, such as edema, inflammation, and pH (Stanisz et al., 2004, Vavasour et al., 2011). NODDI enables the estimation of v_ic_ and v_iso_ using clinically feasible acquisition protocols; however, this comes at the price of making some strong assumption about the intracellular diffusivity (Zhang et al., 2012). In addition, the estimation of v_iso_ can be biased in the white matter (Bouyagoub et al., 2016), and such a bias can affect the estimated AVF. With respect to this study in particular, a further limitation is that the resolution of the 2 techniques was not matched, potentially leading to misregistration and partial volume effects.

## 5 Conclusions

In conclusion, we have shown that the g-ratio varies throughout the life span with a tendency to increase. The corresponding variation in AVF and MVF support the hypothesis that a subtle but consistent reduction in myelin occurs throughout adulthood, whereas axon density follows a more complex pattern on initial increase followed by a decrease. As this is a cross-sectional study, any “change” with age is of course only inferred and longitudinal studies are needed to confirm these findings, as well as address the exciting hypotheses generated by these data.

## Disclosure statement

The authors have no conflicts of interest to disclose.

## Figures and Tables

**Table 1:** Volume [mm^3^] of white matter tracts

Tract	Side	Mean	SD	Range	
ATR	R	6372.302	1059.814	4139.648	9654.785
	L	5526.817	1148.663	3603.516	8222.168
CST	R	5857.101	662.819	4407.715	7092.773
	L	5291.247	592.1852	4091.309	6697.266
Cingulum	R	1505.011	357.8177	224.1211	2078.613
	L	539.3709	203.9112	153.8086	927.2461
Cingulum HP	R	237.6516	82.47427	215.332	435.0586
	L	394.5826	119.1907	101.0742	628.418
Forceps major		6358.655	1186.431	3331.055	8674.805
Forceps minor		18,810.33	2568.761	13,249.51	25,659.67
IFOF	R	7241.147	1120.903	5453.613	10,331.54
	L	7797.055	1261.921	5124.023	11,025.88
ILF	R	6721.898	1000.217	4473.633	8767.09
	L	4655.543	810.1528	2997.07	6626.953
SLF	R	8935.585	1137.081	6886.23	11,153.32
	L	7309.493	891.228	5172.363	9017.578
SLF HP	R	1388.325	290.4695	716.3086	2056.641
	L	679.9959	180.6508	193.3594	1107.422

Key: ATR, anterior, thalamic radiation; CST, cortico-spinal tract; HP, hippocampal portion; IFOF, inferior fronto-occipital fasciculus; ILF, inferior longitudinal fasciculus; L, left; R, right; SD, standard deviation; SLF, superior longitudinal fasciculus.
