# Supplementary material for: Characterizing axonal myelination within the healthy population: a tract-by-tract mapping of effects of age and gender on the fiber g-ratio
Source: Neurobiol Aging. 2017 Jan;49:109–18. doi: 10.1016/j.neurobiolaging.2016.09.016 (PMC5156474; doi:10.1016/j.neurobiolaging.2016.09.016)
Supplement: Supplementary Fig. 1 [file mmc1.pdf]

Supplementary Figure

Example of Registration between MVF and AVF maps

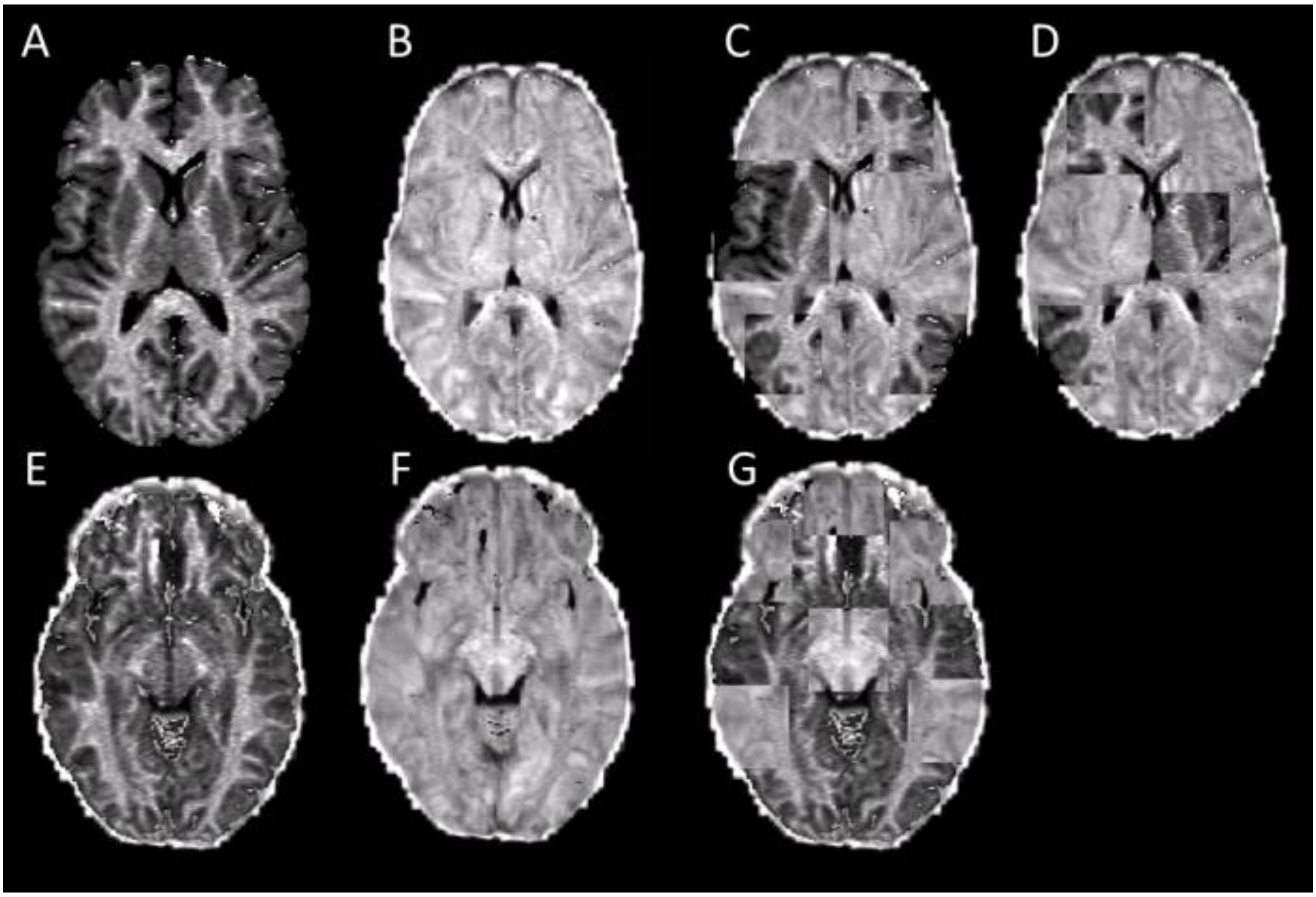

The figure shows two axial sections of MVF (panels A and E) and AVF (B and F) for a participant chosen at random. The goodness of registration can be appreciated from panels C-D and G, where instances of MVF have been overlaid onto AVF map.
